# Feasibility and efficacy of a decision aid for emergency department patients with suspected ureterolithiasis: protocol for an adaptive randomized controlled trial

**DOI:** 10.1186/s13063-021-05140-9

**Published:** 2021-03-10

**Authors:** Elizabeth M. Schoenfeld, Kye E. Poronsky, Lauren M. Westafer, Brianna M. DiFronzo, Paul Visintainer, Charles D. Scales, Erik P. Hess, Peter K. Lindenauer

**Affiliations:** 1 Department of Emergency Medicine and Institute for Healthcare Delivery and Population Science, University of Massachusetts Medical School – Baystate, Springfield, MA USA; 2 Department of Medicine, and Institute for Healthcare Delivery and Population Science Epidemiology and Biostatistics Research Core, University of Massachusetts Medical School – Baystate, Springfield, MA USA; 3 Duke Clinical Research Institute and Division of Urologic Surgery, Duke University School of Medicine, Durham, NC USA; 4 Department of Emergency Medicine, Vanderbilt University Medical Center, TN Memphis, USA; 5 Institute for Healthcare Delivery and Population Science, University of Massachusetts Medical School – Baystate, Springfield, MA USA; 6 Department of Medicine, University of Massachusetts Medical School – Baystate, Springfield, MA USA; 7 Department of Quantitative Health Sciences, University of Massachusetts Medical School, Worcester, MA USA

**Keywords:** Shared Decision-Making, Kidney stones, Medical imaging, Computed tomography, Ultrasound, Randomized controlled trial

## Abstract

**Background:**

Approximately 2 million patients present to emergency departments in the USA annually with signs and symptoms of ureterolithiasis (or *renal colic*, the pain from an obstructing kidney stone). Both ultrasound and CT scan can be used for diagnosis, but the vast majority of patients receive a CT scan. Diagnostic pathways utilizing ultrasound have been shown to decrease radiation exposure to patients but are potentially less accurate. Because of these and other trade-offs, this decision has been proposed as appropriate for Shared Decision-Making (SDM), where clinicians and patients discuss clinical options and their consequences and arrive at a decision together. We developed a decision aid to facilitate SDM in this scenario. The objective of this study is to determine the effects of this decision aid, as compared to usual care, on patient knowledge, radiation exposure, engagement, safety, and healthcare utilization.

**Methods:**

This is the protocol for an adaptive randomized controlled trial to determine the effects of the intervention—a decision aid (“Kidney Stone Choice”)—on patient-centered outcomes, compared with usual care. Patients age 18–55 presenting to the emergency department with signs and symptoms consistent with acute uncomplicated ureterolithiasis will be consecutively enrolled and randomized. Participants will be blinded to group allocation. We will collect outcomes related to patient knowledge, radiation exposure, trust in physician, safety, and downstream healthcare utilization.

**Discussion:**

We hypothesize that this study will demonstrate that “Kidney Stone Choice,” the decision aid created for this scenario, improves patient knowledge and decreases exposure to ionizing radiation. The adaptive design of this study will allow us to identify issues with fidelity and feasibility and subsequently evaluate the intervention for efficacy.

**Trial registration:**

ClinicalTrials.gov NCT04234035. Registered on 21 January 2020 – Retrospectively Registered

**Supplementary Information:**

The online version contains supplementary material available at 10.1186/s13063-021-05140-9.

## Administrative information

The order of the items has been modified to group similar items (see http://www.equator-network.org/reporting-guidelines/spirit-2727-statement-defining-standard-protocol-items-for-clinical-trials/).

**Table:** 

Title {1}	Feasibility and efficacy of a decision aid for emergency department patients with suspected ureterolithiasis: protocol for an adaptive randomized controlled trial
Trial registration {2a and 2b}.	ClinicalTrials.gov - NCT04234035
Protocol version {3}	Protocol Version 2 10/2019
Funding {4}	Agency for Healthcare Research and Quality (AHRQ) 1 K08 HS025701-01
Author details {5a}	Department of Emergency Medicine, University of Massachusetts Medical School – Baystate, Springfield, MA Institute for Healthcare Delivery and Population Science at Baystate Medical Center, Springfield, MA, USA Department of Emergency Medicine, Vanderbilt University Medical Center, Memphis, TN, USA
Name and contact information for the trial sponsor {5b}	Sponsor: Baystate Health, Office of Research Peter D. Friedmann, MD, MPH Chief Research Officer and Associate Dean for Research 3601 Main Street, 3rd Floor, Springfield, MA 01199 Phone: 413-794-7717; Fax: 413-794-0300 Susan.Decelle@baystatehealth.org
Role of sponsor {5c}	Protocol is for an investigator-initiated study. The sponsor played no part in study design; collection, management, analysis, and interpretation of data; writing of the report; and the decision to submit the report for publication.

## Introduction

### Background and rationale {6a}

Ureterolithiasis is common, painful, and costly, but usually self-limited. Every year, emergency departments (EDs) in the USA see over 2 million patients presenting with signs and symptoms of ureterolithiasis [1, 2]. Although the pain from passing a kidney stone is known to be severe, less than 1 in 5 of patients with confirmed stones will require urologic intervention over the course of their disease process, as most kidney stones pass spontaneously within several weeks [2–4].

The gold standard for the diagnosis of ureterolithiasis is non-contrast CT scan—both for its accuracy in diagnosing ureterolithiasis and its ability to pick up dangerous alternative diagnoses [5]. However, over the past decade, evidence suggesting that routine use of CT for the diagnosis of uncomplicated (non-infected) kidney stones is unnecessary has mounted. First, the rate of serious alternative diagnoses is lower than previously believed when older patients and patients with clinically apparent risk factors are excluded [6, 7]. Second, a risk score that stratifies patients based on the likelihood that their pain is caused by a kidney stone, the STONE score, has been developed and externally validated [8, 9]. Use of the STONE score improves clinicians’ pre-test probability for kidney stones and informs the use of ultrasound (US) as a diagnostic alternative to CT [8, 9]. Third, a multicenter randomized controlled trial demonstrated that an US-first algorithm is as safe as a CT-first algorithm and resulted in lower cumulative radiation to patients [10].

While the superior accuracy of CT is not debatable, concerns regarding lifetime radiation exposure have led many to propose alternative diagnostic algorithms [11–13]. Many patients with kidney stones undergo two or more CT scans during the management of each unique episode of colic; fluoroscopy is often used during the treatment of stones; and recurrent episodes of colic, which occur in > 50% of patients, result in repeat imaging [14, 15]. The medical literature demonstrates clearly that patients often do not understand that they are being exposed to ionizing radiation or that this exposure may increase their future risk of cancer [16]. Emergency physicians report a desire to convey this information, but are also not always correct in their own understanding of the risks of radiation [16].

As the decision of whether to utilize CT scanning in the diagnosis of ureterolithiasis has trade-offs involving radiation, accuracy, cost, and time spent in the ED, many feel that it is appropriate for shared decision-making [17]. Shared Decision-Making (SDM) is a process by which clinicians share the risks and benefits of the options and the patients share their values and preferences, and together come to a decision [18].

### Objectives {7}

In this paper, we describe the rationale and methods used to design and test the feasibility and efficacy of the Kidney Stone Choice decision aid in a randomized controlled trial. We hypothesize that the use of the decision aid will significantly increase patient knowledge and engagement and decrease radiation exposure for this population.

### Trial design {8}

**Fig. 1:**
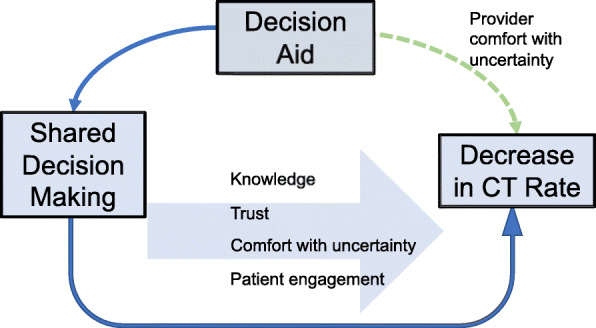
Theoretical model for study design and outcome selection

This pragmatic, adaptive randomized controlled trial compares an intervention group receiving a structured, risk-stratified decision aid, “Kidney Stone Choice (KSC),” to a control group receiving usual care [19]. IRB approval has been obtained and the trial has been registered at www.ClinicalTrials.gov (NCT04234035). Trial design is based on the theoretical model in Fig. 1: we hypothesize that the KSC decision aid may directly decrease CT scan usage via its effects on providers, or may decrease CT scan usage via Shared Decision-Making’s effects on knowledge, trust, and patient engagement. The adaptive design is explained in Outcomes {12}, Sample size {14}, and Fig. 3.

#### Decision Aid Development

Using evidence-based stakeholder engagement methods, we developed a decision aid (KSC) to facilitate SDM in the scenario of suspected ureterolithiasis [20]. The initial decision aid was developed and beta-tested in a single-center decision-aid development study at Baystate Medical Center, Springfield, MA, USA, and details of this process have been previously published [20]. Development of the decision aid iteratively involved patient and clinician stakeholders and resulted in a tool that meets International Patient Decision Aid Standards [21, 22]. The refined 6-page decision aid is available as Supplementary material 1.

Pilot testing demonstrated both feasibility and acceptability in 10 clinician-patient dyads. The decision aid is designed to facilitate, not replace, a conversation between the clinician and the patient. It describes the decision at hand, background information about kidney stones that both clinicians and patients felt was important, and the risks and benefits of the two options (“Wait and see” or “CT before going home”). The goals of the decision aid are to increase patient knowledge about the risk of radiation, increase patient engagement in decision-making, and decrease radiation burden in this population.

## Methods: Participants, interventions, and outcomes

### Study setting {9}

Patients and clinicians will be recruited from the emergency department at Baystate Medical Center in Springfield, MA, USA. This ED sees > 115,000 visits per year, and is staffed by board-certified Emergency Physicians, advanced practitioners, and emergency medicine residents. The trial will be conducted within the flow of routine patient care.

### Eligibility criteria {10}

**Fig. 2:**
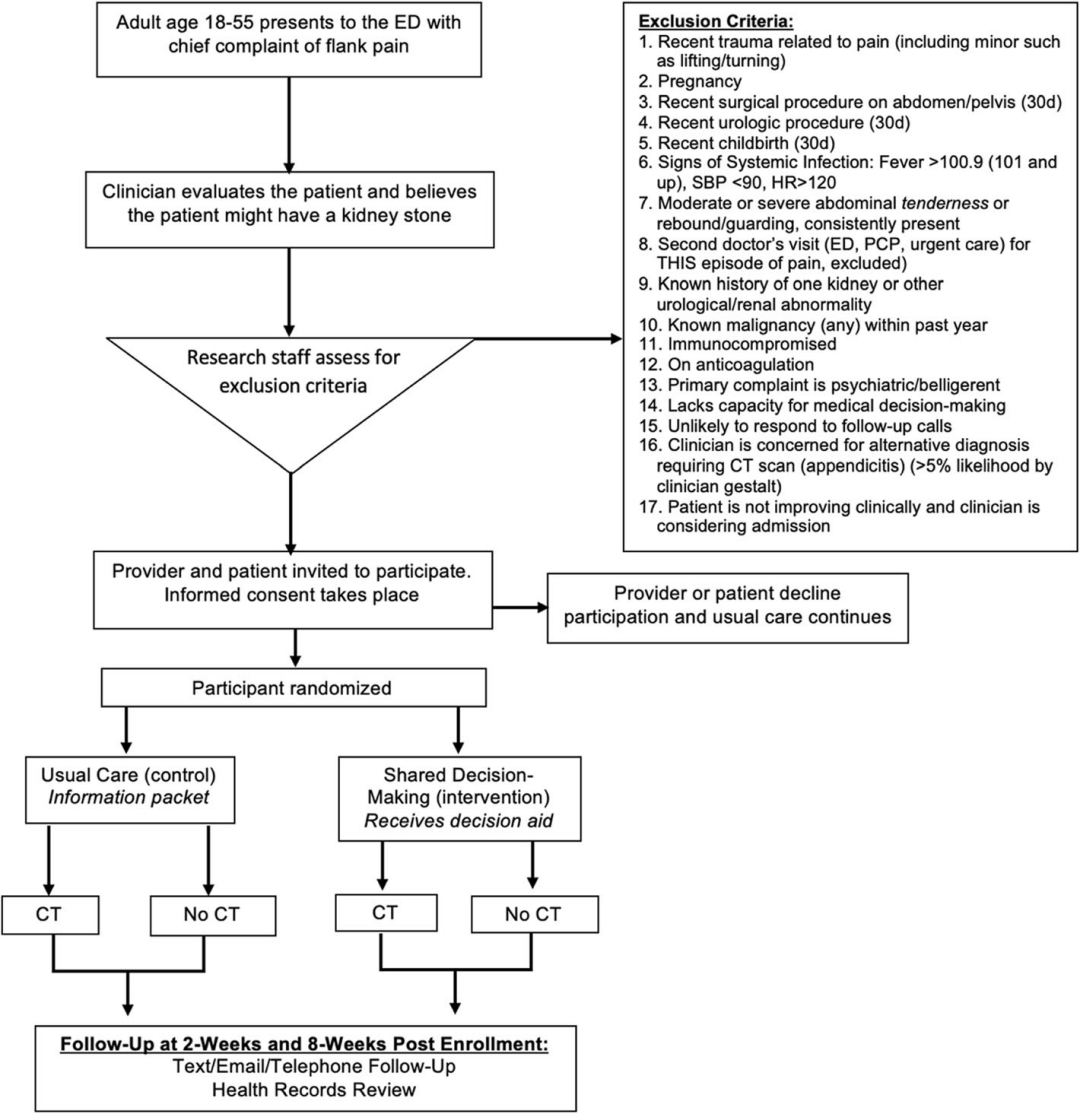
Flow diagram showing the integration of study procedures in the flow of patient care

Eligible patients will include adults age 18–55 presenting to the ED with a chief complaint of flank pain who are being considered by the treating clinician for a CT scan for the diagnosis of ureterolithiasis. Exclusion criteria are listed in Fig. 2. Data will be collected on all patients assessed for eligibility and we will report the number of excluded patients, the rationale for each exclusion, and the number of patients who decline consent to participate in the study in accordance with the CONSORT guidelines for reporting randomized trials [23]. Participants with and without a history of ureterolithiasis will be eligible, but the intervention will be tailored to their history. Eligibility criteria were determined by stakeholder engagement during the process of decision aid development [20].

### Who will take informed consent? {26a}

A study coordinator will identify potentially eligible patients based on the chief complaint and, in collaboration with the treating clinician, confirm patient eligibility for the trial (Fig. 2). The study coordinator will then obtain written informed consent from the patient. Verbal consent will be obtained from participating clinicians at the time of patient consent—if clinicians agree to be recorded. Patients will be consecutively enrolled five to seven days per week whenever a study coordinator is available for enrollment.

Consent for audio recordings will be separate from the consent for the trial and lack of consent for recording will not preclude participation in the trial. Clinicians will verbally consent to recording as well—if either the patient or the clinician does not consent, the conversation will not be recorded. If no conversation takes place regarding diagnostic options, a recording will not be made.

#### Integrating patient recruitment, consent, and delivery of the intervention in the flow of patient care

Figure 2 shows how the process of patient recruitment, consent, randomization, and delivery of the intervention will integrate with the flow of patient care. We will assess patient eligibility and obtain consent shortly after the clinician has evaluated the patient, treated their pain, and begun diagnostic testing (but prior to ordering a CT scan). Patients will then be randomized and receive the intervention or usual care. Patients for whom an exclusion criterion becomes apparent post-randomization (ex. patient who develops a fever, has a positive pregnancy test, or is found to have a solitary kidney on ultrasound) will be classified as screen failures and not analyzed [24].

Patients who are randomized to the decision aid arm will then be given the decision aid, and the clinician will be prompted to discuss the diagnostic options. For the purposes of blinding, patients randomized to usual care will receive a paper informational sheet and the clinician will be prompted to proceed with usual care regarding diagnostic imaging.

## Interventions

### Explanation for the choice of comparators {6b}

This study compares a decision aid designed to facilitate Shared Decision-Aid with “Usual Care,” operationalized as a standard information sheet about nephrolithiasis. Decision aid development has been published previously [20].

### Intervention description {11a}

#### Intervention arm

##### Training

Trainings for practicing clinicians will be provided both via a one-hour lecture prior to the start of the trial and a 3-min “just in time” refresher will be administered just before the SDM conversation. All practicing clinicians will be invited to participate.

##### Certainty (“risk”) assessment and delivery of the intervention

The study coordinator will collect pre-intervention variables from patients, including the components of the STONE score [8]. The coordinator will collect and collate relevant variables from the clinician, including ultrasound results. The clinician will receive a document clarifying the patient’s STONE score and the meaning of that score (Ex. Moderate STONE score + mild hydronephrosis = 90% chance of stone) (see Supplementary Material 2). The study coordinator will then provide the clinician with a color copy of the decision aid to provide a concise refresher of the content. There are two areas of the decision aid where the clinician will be prompted to enter data personalized to the patient. First, they will be prompted to fill in a line about pre-test probability, based on the STONE Score and the ultrasound results. Second, they will be given the option of indicating their recommendation prior to the conversation. The decision aid will then be given to the patient, and the study coordinator will give the patient at least 15 min to read the decision aid prior to discussion with the clinician. The treating clinician will then, using the KSC decision aid as a tool to facilitate a conversation, educate the patient regarding the rationale for their clinical suspicion up to that point in the ED visit and engage the patient in a shared decision regarding whether to obtain a CT at that time.

##### Usual care

Participants in the usual care group will receive an informational intervention. This two-page pamphlet contains relevant information about kidney stones developed as part of the decision aid development process, but does not address decision-making or the use of CT scans. Clinicians who have a patient in the usual care group will be encouraged to continue care as usual. Stakeholder engagement has suggested usual care may include the use of CT, ultrasound, and/or shared decision-making. The usual care group will not have access to the decision aid.

### Criteria for discontinuing or modifying allocated interventions {11b}

The patient or treating clinician may withdraw from the trial at any time. The treating clinician may engage in Shared Decision-Making without accessing the decision aid (if in the usual care arm) or may not engage in Shared Decision-Making despite the use of the decision aid (if in the intervention arm). Subjects for whom an exclusion criteria becomes apparent after randomization (such as a new onset fever or newly discovered pregnancy) will be excluded from analysis and further study procedures and considered screening exclusions.

### Strategies to improve adherence to interventions {11c}

Fidelity to the intervention will be assessed via recording of conversations, if both participants and clinicians consent. Study staff will score in real time conversations that are not recorded using the OPTION-5 (*observing patient involvement*) scale [25].

### Relevant concomitant care permitted or prohibited during the trial {11d}

Clinical care will proceed as directed by treating clinicians. This will include various types of imaging, treatment, and follow-up plans. Department guidelines suggest the use of NSAIDS, anti-emetics, and tamsulosin upon discharge.

### Provisions for post-trial care {30}

No post-trial care is planned. Follow-up is via standard of care: primary care physicians and urology. Patients without primary care physicians will be given phone numbers for referral to primary care. The study site has 24-h urology on-call and available for all follow-up.

### Outcomes {12}

#### Outcome measures: feasibility and fidelity

**Fig. 3:**
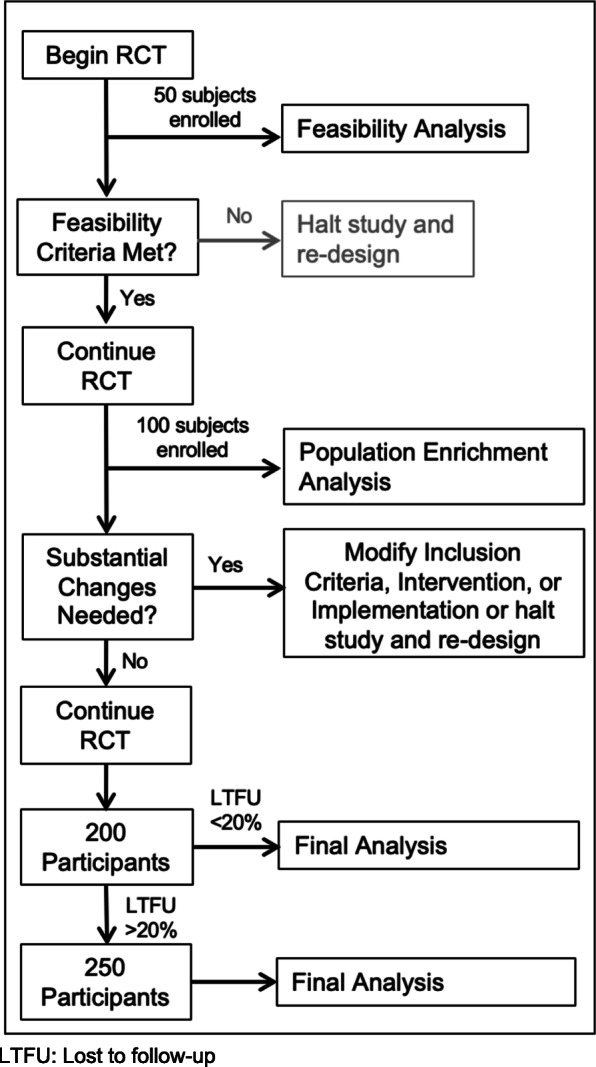
Adaptive design: interim analyses

As this protocol is for an adaptive design randomized trial, we intend to analyze outcomes at two points during the trial. The first interim analysis will examine only feasibility and fidelity to treatment arm (Fig. 3) and will occur after 50 participants are enrolled. Audio recordings of the conversation will be obtained to assess fidelity to the use of the decision aid (in the intervention group) and to monitor for contamination in the control group.

Recruitment will be considered feasible if > 3 participants are enrolled per month and the follow-up rate is at least 70%. Acceptability will be measured by a 7-point Likert scale, and the acceptability goal will be having > 50% of clinicians reporting the decision aid to be somewhat or extremely helpful. Regarding fidelity, a 3rd party will rate conversations on pre-defined criteria for SDM (OPTION-5 scale: *observing patient involvement*) [25]. If > 50% of intervention group conversations and < 30% of usual care conversations are rated as utilizing SDM, this condition will be considered satisfied. If two of three initial goals are not met (recruitment, acceptability, and fidelity), we will pause the trial and implement appropriate changes to the protocol.

#### Outcome measures: efficacy

We selected outcome measures via input from the Steering Committee, patients, and practicing clinicians as well as previous literature [26]. We also have included in the post-enrollment survey a measure assessing outcome priorities in the studied population. As stakeholders generally represent two different groups (patients and clinicians), we considered two outcomes as primary [27]. Clinicians endorsed radiation exposure as the most important primary outcome, measured as the proportion of patients receiving a CT scan during the ED visit and within 60 days and total radiation exposure in 60 days. Patients endorsed knowledge as a primary outcome, and the steering committee created and piloted a 10 question knowledge test.

We also selected a number of secondary outcomes based on stakeholder input. Secondary efficacy outcomes include 1 - clinician effort to engage patients in SDM (OPTION-5 scale), 2 - patient engagement as measured by CollaboRATE and SDM question(s), 3 - patient satisfaction (items from the Hospital Consumer Assessment of Healthcare Providers and Systems [HCAHPS]), 4 - trust in physician (trust in physician scale, 5 questions) and several implementation outcomes regarding the helpfulness of the intervention (see post-encounter patient and clinician data collection forms, Supplementary Materials 3 and 4) [25, 28, 29].

Safety outcomes include the primary outcome of radiation exposure (number of CT scans obtained on day 0 and within 60 days), total radiation in 30 days/60 days (milli-gray [mGy]) from diagnostic imaging, and the incidence of high-risk diagnoses with complications (HRDwC) that could be related to missed or delayed diagnoses. These HRDwC have been previously defined [10].

Operational outcomes will include (1) ED revisits (in 7/30/60 days) and hospital admissions on second visit, (2) ED length of stay (LOS) (minutes), (3) admission rate, (4) final diagnosis for initial visit, (5) 60-day alternative diagnoses, and (6) urologic procedures (in 30/60 days).

Implementation outcomes will include (1) clinicians’ ratings of conversation/decision aid (“would you recommend,” “would you use again,” and qualitative feedback), (2) acceptability (clinicians’ ratings, qualitative and quantitative), and (3) Fidelity (RA checkboxes) as well as adherence to protocol.

### Participant timeline {13}

Enrollment, randomization, and the intervention (or usual care) will occur on day 0. Follow-ups will occur at approximately 14 days and 60–75 days, allowing for multiple attempts at contact up to 16 weeks. Follow-up can occur by text, email, or phone, per patient preference (Table 1).

**Table 1:** Time schedule of data collection and study procedures

Procedure	Screening/enrollment (day 0)	Follow-up 1 (day 14)	Follow-up 2 (day 60–75)
Informed consent	X		
Demographics	X		
Medical History	X		
Randomization	X		
Intervention	X		
Patient knowledge test	X		
Patient engagement survey	X		
Trust in Physician Scale	X		
SDM measures	X		
Clinical outcomes (ED diagnosis, HRDwC, ED revisits, 30/60 day CT usage, admission rate, urologic procedures, primary care visits)	X	X	X
Patient Satisfaction survey	X		
Clinicians’ perspectives assessment	X		

*HRDwC* High risk diagnosis with complications

### Sample size {14}

As per Fig. 3, three discrete analysis times are planned.

Our sample size for phase I (until interim analysis 1) is based on the expert recommendation that at least 50 subjects be included to examine the pragmatics of recruitment [30].

For the endpoint of reduction in the use of CT scans, assuming a base proportion of 80% (currently, the usage rate for kidney stones), a sample of 200 patients (100 patients per group) will provide 87% power to detect a reduction of CT use to 60%, as significant at a *p* value of 0.047 (adjusted to account for the interim efficacy analysis—see explanation below). A final sample size of 200 is large enough to account for a loss of 10% and still provides about 80% power to detect the hypothesized difference. If lost to follow-up rates are higher, a sample size of 250 will be used (125 patients per group).

For the endpoint of knowledge, we hypothesize that an improvement of 2 points (of 10) is clinically significant. If the decision aid improves knowledge from 6/10 to 8/10, we will be powered at 0.80 to detect this with a total of 70 participants.

### Recruitment {15}

Currently, internal data suggests that > 300 patients per year meet inclusion criteria at our primary site, and research staff will be available to approach approximately 1/3 of those who meet criteria. If recruitment is slower than expected, we will expand the length of the trial or enroll participants at one of our 5 community sites. We may also be able to alter research staffing to increase availability of staff during off hours (nights and weekends).

## Assignment of interventions: allocation

### Sequence generation {16a}

#### Randomization

Patients will then be randomized via permuted blocked randomization [31, 32]. Randomization will be stratified by “history of stones” with separate randomization for subjects with a history of kidney stones, as the decision aid for these patients is different, because guidelines suggest avoiding CT for young healthy patients with a history of kidney stones [33]. Allocation will be based on a 1:1 ratio between the intervention and usual care arms. Though we will not be able to blind clinicians to use of the decision aid, patients will not be aware of whether they are receiving usual care or the decision aid, as the consenting information will refer to paper-based information—which accurately describes both the decision aid and the usual care information packet. The research staff will maintain a database recording randomization, which will be separate from general subject-level data and will not be accessible to the PI or statisticians until planned interim analyses.

### Concealment mechanism {16b}

Groups of 4–10 pre-composed opaque envelops will be ordered via subject number and kept in a locked research office, and the envelopes will be distributed sequentially.

### Implementation {16c}

The PI and Research Coordinator will generate pre-composed opaque envelopes to be used for randomization. Trained research staff will enroll patients and assign participants to interventions based on the order of the envelops.

## Assignment of interventions: blinding

### Who will be blinded {17a}

Participants will be blinded to the specific purpose of the trial and to their allocated group; therefore, all patient-centered outcomes (e.g., knowledge) will be gathered from patients who are blinded to group allocation. The consent will inform them that they were being randomized to one of two “ways to receive information,” without specific mention of the role of the decision aid in decision-making. Clinicians and study staff will not be blinded. Feasibility analysis (recruitment and retention) will be blinded, but fidelity analysis (for fidelity to intervention and contamination) will require analysts to know the treatment group of each participant.

### Procedure for unblinding if needed {17b}

As clinicians and study staff are not blinded, unblinding will not occur.

## Data collection and management

### Plans for assessment and collection of outcomes {18a}

Research coordinators will screen all patients seeking care at the study site who have a chief complaint of “flank pain” or “possible kidney stone.” Data from screened patients will be entered into a secure RedCap online standardized data collection form. (All study data will be collected and managed using *REDCap* electronic data capture tools hosted at Tufts University.)

In addition, working clinicians will be prompted via emails, training, and signs in the ED to contact study staff if they have a patient they deem possibly eligible. Reasons for exclusions will be documented, including reasons the patient was not approached or self-reported reasons they did not consent.

Research staff will confirm and document eligibility prior to informed consent. After written informed consent, they will collect baseline variables such as demographics, characteristics of chief complaint, and results of available ED testing. The research staff will note the study assignment in a separate RedCap database, so that the remainder of the study materials are blinded (e.g., the RA assessing the follow-up will not know the intervention group).

After the intervention or usual care is delivered, research staff will collect outcome data on patient knowledge, satisfaction, trust in physician, and the decision made. Standardized data collection forms will be used or data will be entered directly into the RedCap database. Audio recordings will be made of the conversation, if both patient and clinician consent and a conversation occur. These recordings will be independently rated with the OPTION-5 score by two trained team members who are blind to the intervention group [25].

Participants will be given a healthcare diary and follow-up procedures will be explained (Supplementary Material 5). Participants will be contacted 2 weeks and 8 weeks after their visit via phone, email, or text. A standardized follow-up script will assess safety and healthcare utilization. Events occurring within 8 weeks will be recorded and confirmed via chart review. Participants will be asked to sign an authorization so that records for care taking place outside the hospital system can be obtained. Utilization and safety data will include patient-reported data but will be confirmed with chart review and will include subsequent ED visits, CT scans, admissions, procedures, and diagnoses.

Clinicians will be surveyed after each encounter with a brief paper-based survey. If time allows, several open-ended questions will be asked and the answers will be audio recorded. The questions will assess the clinician’s perception of patient involvement in decision-making, the helpfulness of the information, and the efficiency of the interaction.

### Plans to promote participant retention and complete follow-up {18b}

Participants will be contacted multiple times by text, email, or phone to gather follow-up data and maintain engagements. If participants are lost to follow-up, the hospital medical record system will be used to assess for HRDwC. However, if none are found, or there is no data regarding further care, they will be considered lost to follow-up (LTFU).

### Data management {19}

All data collected will be stored using REDCap (Research Electronic Data Capture) hosted by the Tufts Clinical and Translational Science Institute. Electronic capture data point fields will include range limits to protect against accidental entry of impossible data points. Direct data entry at the time of collection will be utilized when possible, but paper forms will be available in the event of connectivity issues. Paper forms will be managed via standard operating procedures, they will be transported directly to research offices after use and stored in locked cabinets prior to data entry. Data entry will occur as close to data acquisition as logistically possible. Data management will be performed by trained research staff under the supervision of the PI. The PI will not have access to the randomization dataset while the trial is ongoing, other than at pre-specified points.

### Confidentiality {27}

All data collected during the course of the study will be kept strictly confidential and will only be accessed by members of the study team as required by study procedures, or by members of the overseeing IRB or other legally authorized parties. All physical documents will be stored in locked cabinets in locked offices of research staff. All electronic data will be housed in RedCap. Email and text messages will be sent from hospital email accounts and HIPAA compliant apps. Anonymized trial data will be available via the PI once the study is complete.

### Plans for collection, laboratory evaluation, and storage of biological specimens for genetic or molecular analysis in this trial/future use {33}

There will be no collection of biological specimens for future use.

## Statistical methods

### Statistical methods for primary and secondary outcomes {20a}

For the primary outcome, the univariable comparison of study groups on the proportion of CTs obtained will be conducted using Pearson’s chi-square. Univariable comparisons of study groups on questionnaire outcomes (e.g., patient knowledge, patients satisfaction) will be conducted with independent sample t-tests.

#### Multivariable analyses

We will apply ICH-E9 guidelines for statistical analysis and the CONSORT statement for reporting of findings for clinical trials [31, 34]. For multivariable analyses, linear regression or logistic regression will be used to adjust for baseline characteristics that do not appear to be balanced upon inspection or show moderate to large standardized effect sizes. Covariates that do not meaningfully modify estimates of treatment effect or otherwise contribute to clinical interpretation may be removed from the final model.

In multivariable models, we will assess for heterogeneity of treatment effects by including interaction terms (specifically, age, gender, and race/ethnicity, insurance, education, and health literacy) with treatment group [35]. In view of our limited sample size to detect significant interaction terms, we will consider any interaction term as worthy of further evaluation if significance testing achieves a critical test level of 0.15.

Our primary analyses will proceed via an intention-to-treat approach, as some cross-over is expected. All measures of treatment effects (e.g., odds ratios, difference in means or proportions) as well as estimates of group specific effects (e.g., means and proportions) will be reported with 95% confidence intervals. However, patients with exclusion criteria that manifest post-randomization will NOT be included (ex. pregnancy diagnosed in the ED).

### Interim analyses {21b}

The first interim analysis, which will occur after 50 patients are enrolled, will only consider feasibility and fidelity of the protocol (i.e., whether recruitment and retention goals are met, whether the protocol is acceptable to clinicians, and whether the protocol is being administered as planned). There will be no planned assessment of efficacy outcomes at this interim analysis. As such, there will be no adjustment to the *p* value for this interim analysis.

At the second interim analysis (approximately 100 patients evaluable for efficacy), we will evaluate the sample to determine whether further enrichment of the sample is required (e.g., further refinement of inclusion/exclusion criteria, such changing the maximum age or excluding patients with a history of ureterolithiasis). We will assess efficacy as this interim analysis, and therefore will adjust the critical test level accordingly. Using the O’Brien-Fleming approach to the alpha spending function, one evaluable interim look at approximately 50% of the sample, the critical test levels for early termination for efficacy will be *p* ≤ 0.003 and *p* ≤ 0.047, for a two-sided test [36, 37]. That is, if at the first evaluable interim analysis the difference between study groups in both primary outcomes achieves a significance level of *p* ≤ 0.003, the study will stop. The final significance test will be conducted at *p* ≤ 0.047, rather than *p* ≤ 0.05.

Initial data analysis will describe the distribution of all outcomes by treatment group. Continuous measures will be reported using means, standard deviations, medians, and percentiles. Categorical measures will be reported using frequencies and percentages. Rather than testing baseline covariate imbalance, stratified analyses will be conducted for baseline covariates that are known to be strongly associated with outcomes, as well as those that show imbalance on inspection [38].

### Methods for additional analyses (e.g., subgroup analyses) {20b}

Due to anticipated cross-over, we will also assess for contamination, as it is expected that some proportion of the usual care group will have elements of a Shared Decision-Making conversation with their clinician. Contamination will be measured via two methods: self-report by clinicians (“Did you have a SDM conversation with the patient?”) and 3rd party assessment via the OPTION-5 scale [25]. As a secondary analysis, a per-protocol analysis will examine efficacy. That is, treatment received (per-protocol) will be determined by research staff assessment, and a per-protocol analysis will be performed. Per CONSORT guidelines, this will be presented in parallel with the intention to treat analysis [39]. As SDM may be delivered in the usual care group, but without the decision aid, we expect that a per-protocol analysis may results in three distinct groups: one – randomized to usual care and did not received SDM, two – randomized to usual care and received SDM (without the decision aid), and three – randomized to SDM and received both SDM and the decision aid.

### Methods in analysis to handle protocol non-adherence and any statistical methods to handle missing data {20c}

As noted above in additional analysis, a secondary, per-protocol analysis will examine outcomes by whether or not the participant was engaged in Shared Decision-Making, as reported by clinicians and judged by study staff, regardless of whether the decision aid was used.

Missing data will be described and, if extensive, limitations will be identified in our discussion. We will compute standardized effect sizes to help determine the degree of imbalance on baseline characteristics and subsequently inform multivariable model development.

### Plans to give access to the full protocol, participant level-data and statistical code {31c}

De-identified data will be available from the PI upon request at the completion of data analysis.

## Oversight and monitoring

### Composition of the coordinating center and trial steering committee {5d}

The Primary Investigator and Co-Investigators will oversee the conduct of the trial. The Steering Committee has been previously described and is made up of clinicians, patients, and community members [20]. The research staff within the Department of Emergency Medicine will oversee data management, and the Biostatistics Core will oversee the analysis. The Steering Committee will act as an endpoint adjudication committee, as needed.

### Composition of the data monitoring committee, its role and reporting structure {21a}

A Data Safety Monitoring Plan (DSMP) is in place and included separately (Supplementary Material 6). As this study is minimal risk, it does not require a full independent DSMB. However, the DSMP utilizes external physician reviewers for all Serious Adverse Events and clearly delineates definitions of adverse events and stopping rules. The study will be paused and independently reviewed if 3 “Serious, Moderate/Severe” SAEs determined to be “probably related” are reported. Specific details are listed in the DSMP.

### Adverse event reporting and harms {22}

Adverse events (AE) will be collected with routine data at scheduled follow-up. As defined in the Data Safety Monitoring Plan, AEs will include (1) admission at the time of enrollment or within 30 days, (2) inpatient surgical procedure, and (3) death. As both admission and surgical procedures are expected, serious adverse events (SAE) are defined as hospitalization for a non-urologic procedure or antibiotics, anticoagulation, or other medications, admission to the ICU, or death. For the purposes of this study, hospitalization for pain control or a urologic procedure will not count as a SAE, as hospitalization is an expected outcome in 10% of patients with renal colic.

### Frequency and plans for auditing trial conduct {23}

All AEs will be reviewed by an independent physician. SAEs will be reported to the IRB within 72 h of study team discovery. The IRB will perform audits as per standard operating procedures. The independent physician and IRB are both independent from the investigators and the sponsor.

### Plans for communicating important protocol amendments to relevant parties (e.g., trial participants, ethical committees) {25}

The local IRB will evaluated amendments prior to any major changes to the protocol. If approved, ClinicalTrials.gov will be updated about any major changes. If changes require reconsent of participants, based on IRB standard operating procedures, participants will be contacted and re-consented.

### Dissemination plans {31a}

Study results will be disseminated via relevant conferences, peer-reviewed publications, and standard social media discussion. No publication restrictions exist.

## Discussion

We have described the methodology for an adaptive-design, single-center randomized controlled trial to measure the feasibility, fidelity, efficacy, and effectiveness of a decision aid on patient-centered outcomes, safety, and healthcare utilization. Only three decision aids have been designed and tested in the setting of ED care; this study incorporates lessons learned from previous studies and utilizes an adaptive design for more efficient study conduct [26, 40].

It is necessary that this study begins with a feasibility assessment. Our previous work has shown that ED clinicians are not consistently aware of or impacted by SDM research [41]. This could translate to a barrier to enrollment or random group allocation, which can be assessed in the feasibility portion of this study.

For a reliable assessment of efficacy, randomization is necessary. Previous studies of SDM interventions have randomized at either the patient or the clinician level [40]. Although randomizing at the clinician level could theoretically reduce contamination, the challenges of clinician randomization at an academic center (where care is delivered by teams including a resident, advanced practice provider, and attending physician) made this option problematic. Additionally, care for the comparison group will not be dictated, so “usual care” may vary. However, the decision aid will not be available to usual care clinicians who choose to discuss imaging options with their patients. In some ways, this will lead to three groups: usual care (no SDM), usual care (SDM but no decision aid), and SDM delivered with a decision aid. The per-protocol analysis will help tease out the effects of the decision aid versus the effects of SDM delivered without the decision aid.

In conclusion, this trial will examine the feasibility and compare the efficacy of a decision aid to usual care in the diagnostic workup of patients with suspected kidney stones. By designing and conducting an adaptive trial, we will efficiently assess feasibility and create opportunities for flexibility and sample enrichment. If our hypotheses are supported, our results will help clinicians involve patients in diagnostic decision-making and pave the way for a multicenter trial to test generalizability of the decision aid.

## Trial status

Under protocol version 2, recruitment began in December 2019. We expect recruitment to last 24–36 months.

## Supplementary Information

**Additional file 1.** Decision aid. A 6-page decision aid with illustrations.

**Additional file 2.** STONE Study Decision Aid Guidelines. A 3-page decision aid guidelines for clinicians.

**Additional file 3.** Post-encounter patient data collection forms, 4 pages.

**Additional file 4.** Post-encounter patient feedback about clinician data collection forms, 6 pages.

**Additional file 5.** Use of Healthcare Services. A 4-page healthcare procedures diary.

**Additional file 6.** Data Safety Monitoring Plan and Instructions, 4 pages.

## Abbreviations

SDM: Shared Decision-Making
ED: Emergency department
CT: Computed tomography
US: Ultrasound
IRB: Institutional Review Board
DSMP: Data and Safety Monitoring Plan
RA: Research Associate
RC: Research Coordinator
HRDwC: High risk diagnosis with complications
OPTION-5 Scale: Observing patient involvement
HCAHPS: Hospital Consumer Assessment of Healthcare Providers and Systems (survey)
RCT: Randomized controlled trial
LTFU: Lost to follow-up
AE: Adverse event
SAE: Serious adverse event
ICU: Intensive care unit
