# Supplementary material for: Feasibility and efficacy of a decision aid for emergency department patients with suspected ureterolithiasis: protocol for an adaptive randomized controlled trial
Source: Trials. 2021 Mar 10;22:201. doi: 10.1186/s13063-021-05140-9 (PMC7944622; doi:10.1186/s13063-021-05140-9)
Supplement: Supplementary file 1 — Additional file 1. Decision aid. A 6-page decision aid with illustrations. [file 13063_2021_5140_MOESM1_ESM.pdf]

Supplementary Material 1: The decision aid: “Kidney Stone Choice (KSC)”

We have a decision to make. Together.

Your clinicians want you to be involved in the decisions that affect your body. When you and your clinician make decisions together, this is called, “Shared Decision-Making.”

This pamphlet gives you information about the tests that we use in the Emergency Department, or “ED,” to diagnose kidney stones. After you read through it, you and your clinician will talk about the options. (Your clinician may be a doctor, physician assistant, or nurse practitioner.)

The decision you will discuss with your clinician today is about:

❑ ***CT scan today*** *versus* ❑ ***no CT scan today***

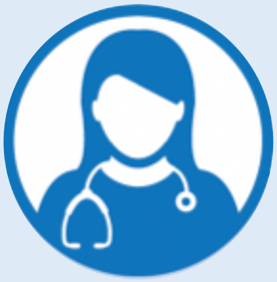

Your clinician has medical expertise. They can answer your questions, based on science and evidence.

You are the expert in your life and your body. You can tell your clinician what is important to you.

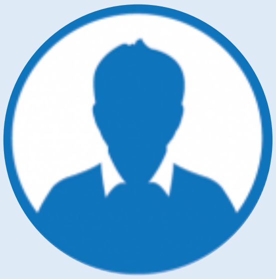

Together, we can make a decision that everyone is comfortable with.

First, it’s important for you to know more about kidney stones.

What Are Kidney Stones?

It looks like you may have a kidney stone.

A kidney stone is a small stone that forms in your urine, right in your kidneys. Stones can be as small as sand or bigger than a pea. Your body tries to pee the stone out, and this causes pain that can be anywhere from your kidneys (back) down to your bladder. Some people feel the pain go down to their groin (or testicles or labia).

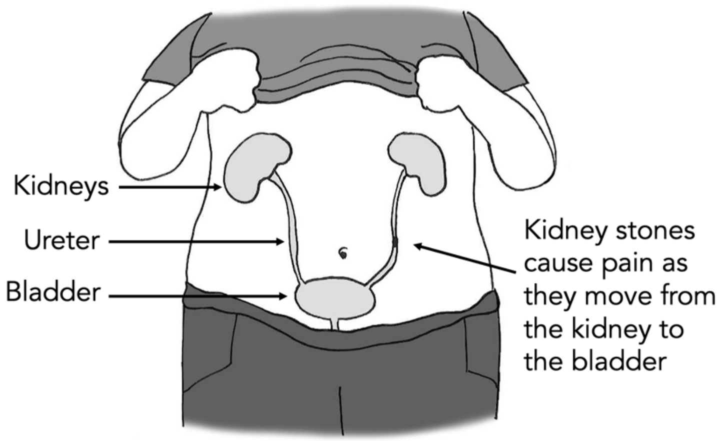

(Feel free to write all over these pages! Questions, notes, check-boxes, or anything!)

Signs of a kidney stone: *(Go ahead and check off your symptoms below)*

- ❑ The pain is severe and started suddenly.
- ❑ The pain comes and goes, and it's hard to sit still. The pain is in your mid- or lower-back, on one side, and might spread around to the front.
- ❑ The pain comes with nausea or vomiting.
- ❑ The pain started recently, such as earlier today (not weeks or months ago).
- ❑ Your urine test shows red blood cells.*
- ❑ Your ultrasound shows some swelling in your kidneys.*

**If you're not sure about these, ask your clinician for your results.*

If you have 4 or more checked, there is a VERY HIGH chance that you have a kidney stone. → → → → → → → → →

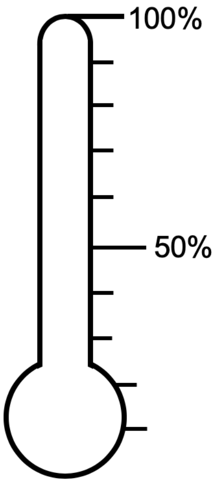

_____%

How do clinicians diagnose kidney stones?

- Most kidney stones come with many of the signs listed above.
- Sometimes people have blood cells in their urine (the urine may look normal).
- Blood tests are usually normal and don't always need to be checked.

The decision to discuss today: Do you need a CT after your ultrasound?

It is recommended that you and your clinician talk about this decision because…

… guidelines recommend starting with an ultrasound

- An ultrasound can give a lot of information about the stone, and sometimes will see the stone.
- There is no radiation from an ultrasound, it can be repeated without concern.
- An ultrasound can also show gallstones, ovarian cysts, kidney stones, and other causes of pain.
- An ultrasound is often faster and cheaper, and is good enough most of the time.
- You can always get a CT later if you are not feeling better.

BUT:

- Some people will still need a CT scan at a later time.

… some guidelines recommend a CT scan

- A CT scan will usually show the size and location of the stone.
- CTs are pretty accurate for things like appendicitis, and some other causes of belly pain.
- CT scans help the urologist (kidney stone doctor) plan for a procedure, if you need one.

BUT:

- A CT scan exposes you to radiation. This radiation is similar to about 200 x-rays. This amount of radiation may increase your future risk of cancer by a very small amount.
- If you have had kidney stones before, guidelines recommend avoiding a CT if possible, because of the additional radiation.

There are risks and benefits to both options, so it's important that you and your clinician make a decision together.

Emergency Department (ED) care for patients with Kidney Stones

Before making a decision, you might want to know more about kidney stones.

**What is a kidney stone?**	• Kidney stones form when minerals like calcium form crystals in the urine.<br>• Stones start to cause pain when they move down into the ureter (the tube that connects the kidney to the bladder).<br>• The good news is that the pain can be treated with medications and **most kidney stones will pass through on their own.**
**Why did I get a kidney stone?**	• Some people get stones because they have been dehydrated or they have too much calcium in their urine. Often, it's just luck and we don't know why one person gets kidney stones.<br>• People who have a stone once are likely to have one again, in the next 5-10 years.
**When will I feel better?**	• Your clinician knows that kidney stones can be very painful and can prescribe pain medications.<br>• Some people pee out the stone in a day or two, but for other people it can take up to 3-4 weeks. During that time, some people need pain medication, and some people need to see a kidney stone specialist.<br>• If you are feeling better in the ED, that is great, but **the pain often comes and goes for a few days (and sometimes weeks) before it is totally gone.**
**What happens in the ED?**	• You will get nausea and pain medications, if needed.<br>• Your team will check your urine for signs of infection.<br>• Your team will see if you have any signs that you need to be admitted to the hospital (like fever or vomiting that does not stop).
**Will I go home, or be admitted?**	• About 9 out of 10 of people with kidney stones feel well enough to go home after treatment in the ED. Only **1 out of 10 will get admitted** (they will have to stay overnight in the hospital).

After the Emergency Department

**Will my stone pass on its own?**	• Most stones pass in the urine without treatment. About 1 out of 10 people like you will need help from a urologist (kidney stone doctor) over the next few weeks to help pass the stone.<br>• Experts agree that most patients should give the kidney stone time to pass on its own. During this period, the doctor will prescribe medicines to relieve pain and to make it easier for the stone to pass.<br>***Stones can take days or weeks to pass, and people can expect episodes of pain during that time. You can take medications as needed for pain.***
**What are the treatments**	• There is a medication that helps your stone pass (Tamsulosin).<br>• If the stone doesn't pass in 2-3 weeks, or if the pain becomes too severe, you should see a doctor to see if other tests or treatments are needed.

**for kidney stones?**	• <u>If you develop a fever</u>, you need to see a doctor immediately. It is recommended that people with a kidney stone and <u>a fever over 101</u> get a CT scan, because they may need a procedure to help the stone pass.<br>• Possible treatments include surgery to remove the stone, or lithotripsy (where shock waves break the stone into smaller pieces that are easier to pass).<br>• These treatments can have side effects like pain, infection, or complications of surgery. This is why it makes sense to give the stone time to pass on its own.
**What medications should I take?**	1. Ibuprofen (like Advil or Motrin) 400-600 mg, 3 or 4 times in 24 hours as needed for pain<br>2. Acetaminophen (Tylenol) 650 mg, 3 or 4 times in 24 hours as needed for pain<br>3. Tamsulosin (Flomax) 0.4mg, once a day until pain is gone<br>4. Other medications for nausea (Ondansetron/Zofran) are taken up to 3 times a day.<br>5. Narcotic pain medications are sometimes prescribed and should be used carefully.
**What else should I do?**	1. Drink plenty of water.<br>2. **Use a strainer to check your urine for the stone.** The stone can be sent for analysis, which might help you learn how to prevent more stones in the future. It will also confirm your diagnosis of a kidney stone.<br>3. Make a recheck appointment with your primary care clinician 7-14 days after your hospital visit. If you don't have a doctor, you can call 413-794-5412 to find a primary care doctor.
**What should I watch out for?**	1. If you have a **fever** (temperature of 101 or higher) or your pain or nausea is not controlled by the medications, you should return to the ED.<br>2. If you are unable to urinate for more than 12 hours, return to the ED.<br>3. If your symptoms continue for more than 2 weeks, you may need to see a urologist (kidney stone doctor) for treatment of the stone. If you have not had a CT scan, you should have a CT scan (ordered by your primary doctor or the ED) before seeing the urologist.<br>4. If you are feeling better, you should talk to your primary care clinician about having an outpatient ultrasound 6 weeks after your ED visit to make sure there are no signs of the stone. This is not necessary if you catch the stone with a strainer.
**Other patients with kidney stones want you to know:**	1. Narcotic pain medications can be addictive, use them sparingly. Other medications like Ibuprofen (Motrin) and Toradol (IV medication) work well for kidney stone pain.<br>2. Stay hydrated! Now that you have had one stone, you are at risk for another stone. The best way to avoid kidney stones is to stay hydrated and eat a healthy diet.

Now that you know all about kidney stones, let's talk about the decision we have to make. Here are the options:

	Plan 1:<br>❑ Wait and See (Delay/Avoid CT)	Plan 2:<br>❑ CT scan before going home
**What would this plan look like?**	If you are feeling better, you could go home with medications and a clear plan from your clinician. Most people will pee out their stone, but if you are still having episodes of pain in 1-2 weeks, you should see your doctor or return to the ED and have a CT scan, and then see a urologist.	You could stay in the ED for a CT scan. This would show all the details of the stone, but it does not usually change what happens next. Most people will go home and pee out the stone, and a few will have to see a urologist (kidney doctor). Some people will end up with multiple CT scans.
**This plan is good for you if:**	❑ You are starting to feel better<br>❑ Your clinician thinks there is a low chance that your pain is caused by something other than a kidney stone<br>❑ You would prefer to minimize radiation to your body *AND/OR* you have had CT scans before	❑ You have a fever<br>❑ Your pain has been going on for more than a week or two<br>❑ Your clinician is worried you might have appendicitis or another infection<br>❑ Your clinician thinks you need to see a urologist (kidney stone doctor) today
**What are the benefits of this plan?**	1. No radiation to your body<br>2. Shorter ED visit<br>3. Lower cost (to you or your insurance)	1. Your clinician would see size of stone<br>2. We could see stone "mimics" (things that act like stones but aren't)
**What are the risks (or disadvantages) of this plan?**	1. Your clinician could miss a diagnosis that acts like a kidney stone but isn't.<br>2. You may still need a CT in the future if you don't feel better in 1-2 weeks.	1. You will be exposed to radiation from the CT scan one or more times. One CT is not a lot of radiation, but people with kidney stones often get multiple CT scans.<br>2. CT scans usually keep you in the ED longer.<br>3. CT scans usually cost you or your insurance more than an ultrasound.<br>4. You may need another CT in the future.

	Summary of the differences of the two options:	
	Plan 1:<br>Wait and See<br>(Delay/Avoid CT)	Plan 2:<br>CT scan before going home
Radiation Exposure	None	Some
Cost	Lower	Higher
Time in the ED	Less	More
Certainty in Diagnosis	50% to 90+%<br>(ask your clinician)	99%

Some things you might want to discuss with your clinician:

Questions you might ask your clinician:	• What were the results of the tests? (Like ultrasound and urine tests)<br>• If I get a CT, will the results of the CT change the plan?<br>• What is the chance that I have a "kidney stone mimic" - something like appendicitis or diverticulitis?<br>• Do you think I need to see a urologist today?
Please tell your clinician:	• What do you feel about these options?<br>• Do you have a primary care clinician you can follow up with?<br>• Are you able to come back to the ED if your pain is not controlled, or if you start having a fever?<br>• Have you had a CT scan before?

What do you think about these options?
Please share your thoughts with your clinician.

Put questions and notes here: ____

____

____

____
