# Supplementary material for: Feasibility and efficacy of a decision aid for emergency department patients with suspected ureterolithiasis: protocol for an adaptive randomized controlled trial
Source: Trials. 2021 Mar 10;22:201. doi: 10.1186/s13063-021-05140-9 (PMC7944622; doi:10.1186/s13063-021-05140-9)
Supplement: Supplementary file 2 — Additional file 2. STONE Study Decision Aid Guidelines. A 3-page decision aid guidelines for clinicians. [file 13063_2021_5140_MOESM2_ESM.pdf]

STONE Study Decision Aid Guideline For Clinicians
SHARED DECISION MAKING group

WHY avoid CT: In our own patient population, we have found that **in our young kidney stone patients, half have already had two CTs, and 20% have had four or more.** This decision aid educates patients about the use of CT scan and nudges them to avoid CT when reasonable.

RISK STRATIFICATION PAGE:

STONE Score

Factor	Descriptor	Points	Your Patient
Sex	Male	3	
	Female	0	
Timing (duration of pain before beginning of ED visit)	<6 hours	3	
	6-24 hours	1	
	>24 hours	0	
Race	Non-black	3	
	Black	0	
Nausea	Vomiting	2	
	Nausea alone	1	
	Neither	0	
Red Blood Cells in Urine	Present	3	
	Absent	0	
Total		13	

Score categories:

0-5 = Low likelihood of stone. These patients have a 10% chance of having a stone. Work-up should be guided by OTHER diagnoses on your differential.

6-9 = Moderate likelihood of stone. These patients have a 50+% chance of having a kidney stone. If any hydronephrosis on ultrasound, this goes up to ~90%.

10-13 = High likelihood of stone. These patients have a 90+% chance of having a stone, even without hydronephrosis.

The higher the likelihood of a stone, the lower the likelihood of a dangerous alternative diagnosis.

Your patient has:

- ❑ A history of kidney stones → indication to avoid CT as possible
- ❑ No history of kidney stones

Your ultrasound results:

❑ Solitary kidney<br>❑ Calyceal rupture (free fluid)<br>❑ Severe hydro (minimal parenchyma)	→	**CT recommended**
❑ No hydro<br>❑ Mild hydro<br>❑ Moderate hydro	→	**CT not indicated**

Based on the above information, your patient has:

❑ 10% chance of kidney stone as cause of pain, unclear likelihood of other pathology	→	**use shared decision-making**
❑ 50% chance of kidney stone as cause of pain, unclear chance of other pathology	→	**use shared decision-making**
❑ >90% chance of kidney stone as cause of pain, <2% chance of other pathology	→	**use shared decision-making <u>with nudge</u>**

*** If your patient spikes a fever or has unrelenting pain requiring admission, they are not appropriate for this study. But be aware that renal colic pain will come and go – so pain may return after you've discussed options.*

SHARED DECISION MAKING is when there are two medically reasonable options, and the clinician helps the patient understand and weigh the options. The patient contributes his/her opinions and they make a decision together. SDM is appropriate here because there are tradeoffs to the two options. ***Many physicians think that avoiding CT is the most appropriate option for a patient with a low likelihood of a dangerous alternative diagnosis.***

FAQs

1. Why do we need this pathway? What's wrong with our current practice?

CT for kidney stones has been identified by ACEP as an overutilized test. The average number of CTs per episode of renal colic is 1.7, and most people will have another stone, and more CT scans.

2. How much radiation is a CT?

Each CT is ~5mSv. Of 500-1000 young adults receiving a CT, one will eventually develop cancer from that radiation. This is a tiny risk for an individual, but on a population level, **medical radiation causes about 30,000 cancers per year in the US.**

3. How does ultrasound fit in this?

- There are 3 findings that generally should lead to CT: solidary kidney, severe hydronephrosis, or calyceal rupture (relative) which is free fluid around the kidney.
- There are several findings that obviate the need for CT: stone visualized in the bladder, stone at the UVJ, or stone visualized elsewhere.
- Mild/moderate hydronephrosis is reassuring for you and the patient that the diagnosis IS stone.
- However, "*no hydronephrosis"* means the patient is at a lower likelihood of needing a procedure, **it does not mean they need a CT for confirmation.**

4. Why did we include people with a history of stones in this study? Do they follow a different path?

Guidelines tell us we should AVOID CT in patients with a history of stones. However, many of these patients do get CT scans. We are including them, but with "the Avoid CT" option checked, to both educate them and see how this decision aid works for this population.

5. Don't I need a CT for planning for a possible procedure?

A trial of spontaneous passage (watchful waiting) is the guideline for all stones <10mm (assuming healthy patient). Only 10% of patients who feel well enough for discharge will need a procedure by one month – if these patients need a CT, they can have one ordered by their PCP or return to the ED as needed.

6. Won't this pathway increase return ED visits?

We don't know. About 15% of kidney stone patients return to the ED anyway, usually because of poor pain control. Increased knowledge and engagement in the plan may decrease future visits, even if no CT is obtained on the first visit.

7. Is it ok for me to give the patient my opinion?

Yes, the decision aid allows for you to check off which decision you think makes the most sense. **However, it's important that you make sure you elicit how the patient feels about the decision – as they may have valid reasons for making an alternative choice.** Additionally, we want them to feel involved in the decision, we do not want it to feel like a force, more like a suggestion.

Quick answers to questions your patients may ask

1. Are you sure it's a kidney stone?

If there is a high STONE score, or even a middle STONE score and hydronephrosis, it's >90% likely to be a kidney stone. What's most important is how the patient is feeling. There is a 2% (or less) chance it is something dangerous, if the patient is young, healthy, and has flank pain, only mild abdominal tenderness, and no fever.

2. What happens next?

We give you medications and you go home – you may have some pain as you try to pass the stone. We'll give you very specific instructions about what to do (Strain urine. If fever, return to the ER. If still having intermittent pain, see your primary care doctor.)

3. Are there other risks that aren't listed here?

A. Another risk to having a CT is something called "Incidental findings." Any time you get a CT, you risk finding something that may not be important, but might require further testing. These small findings, like a lung nodule or a small mass on an adrenal gland, can cost you money and time, and they make many people very worried until the tests are all done.

B. The main risk to NOT having a CT is that you have something like appendicitis, which needs surgery, or something even more unusual. This is why we want you to return if the pain becomes different, severe, or continuous, or if you have a fever.

4. Doc, what would YOU do?

It's ok to give the patient your opinion, but do not just tell them what you want them to do. Instead, explain how different values/feelings point different people in either direction. Example, "Because you are young and healthy and feeling better, it might make sense for you to try to avoid radiation, and come back if you're feeling worse, but if you were still having a lot of pain, or you spiked a fever, it would make sense for you to get a CT scan."

5. What is my chance of needing a procedure?

Patients who are feeling well enough for discharge have a 1 in 10 chance of needing a procedure at 1 month. Therefore, if your patient is still having pain in a few weeks, they should get a CT scan (or any time they spike a fever).
