# Supplementary material for: Feasibility and efficacy of a decision aid for emergency department patients with suspected ureterolithiasis: protocol for an adaptive randomized controlled trial
Source: Trials. 2021 Mar 10;22:201. doi: 10.1186/s13063-021-05140-9 (PMC7944622; doi:10.1186/s13063-021-05140-9)
Supplement: Supplementary file 3 — Additional file 3. Post-encounter patient data collection forms, 4 pages. [file 13063_2021_5140_MOESM3_ESM.docx]

Supplementary Material 3: Post-encounter clinician data collection form

**Statement of Informed Consent and gathering of Informed Consent Documents**

Thank you for taking the time to be part in this survey for research purposes. The purpose of this research is to help us test a decision-aid, a paper tool that patients and doctors can use to help them make a decision. We expect to include about 400 people in this study. The survey will take 1-5 minutes of your time. Your participation in this study is voluntary and your decision will have no impact on your relationship with the researchers, your employer, or with Baystate Medical Center.

The survey does not include questions that are sensitive or personal. You are free to skip any question for any reason. There is a minimal risk in participating in this survey but there is always the risk of others who are here hearing your responses. We will record the following information about you: (below) but we will not collect any personal identifying information such as your name, medical record number, or date of birth. We will protect the confidentiality of your research information by not recording your name or personal information*.* You may not benefit from this study, other than learning about Emergency Department care, but the information gathered from this research may help in improving Emergency Department care.

By agreeing to continue with this survey, you are consenting to participate in this research study with the understanding that you are free to withdraw at any time. If at any time you wish to discontinue your participation, you may simply notify the research staff and leave, and it will not result in any penalty or loss of benefits to which you are otherwise entitled.  *If you are a Baystate employee, you have every right to refuse to participate in this survey. Neither your participation nor your refusal will be communicated to your supervisor, nor will it affect your employment or your performance evaluation.*

If you have any questions about this study, or have a complication or injury that you believe may be related to this study, please contact Elizabeth Schoenfeld, MD at 413-794-2285.

If you would like to discuss your rights as a research participant, or wish to speak with someone not directly involved in this study, please contact Baystate Medical Center Institutional Review Board at (413) 794-4356.

**Post-Encounter Questions for Clinician**

1. Regarding the decision about whether or not to obtain a CT scan, who made this decision?

| ❑ | I made the decision on my own (or with other clinicians). |
| --- | --- |
| ❑ | I made the decision after seriously considering the patient’s opinion. |
| ❑ | The patient and I shared the responsibility for making the decision after considering both of our opinions. |
| ❑ | The patient made the decision after seriously considering my opinion. |
| ❑ | The patient made the decision on his/her own. |

Demographics

2. You are:

| ❑ | Advanced Practice Provider (PA or NP) |
| --- | --- |
| ❑ | Resident |
| ❑ | Fellow |
| ❑ | Attending |

3. Have you had any previous patients in this study (check all that apply):

| ❑ | No |
| --- | --- |
| ❑ | Yes – in usual care group |
| ❑ | Yes – in SDM group |

4. How many previous patients have you had enrolled in this study: _____

**FOR SDM GROUP**

5. Did you use the nudge check box:

❑ No ❑ Yes ❑ I’m not sure

**If yes**, which did you check:

❑ Plan 1 – no CT/watchful waiting

❑ Plan 2 – CT scan today

6. Did you have an opinion about which choice YOU preferred before the conversation?

| ❑ | ❑ | ❑ | ❑ | ❑ | ❑ | ❑ |
| --- | --- | --- | --- | --- | --- | --- |
| I felt NO CT was best for them |  |  | No, I was completely neutral |  |  | I felt a CT NOW was best for them |

7. Would you *recommend* using this decision aid to other providers?

| ❑_7_ | ❑ | ❑ | ❑ | ❑ | ❑ | ❑_1_ |
| --- | --- | --- | --- | --- | --- | --- |
| Yes, I would strongly recommend it |  |  | Not sure whether to recommend it or not |  |  | No, I would strongly recommend against it |

8. How helpful was the paper pamphlet that you used today?

| ❑_7_ | ❑ | ❑ | ❑ | ❑ | ❑ | ❑_1_ |
| --- | --- | --- | --- | --- | --- | --- |
| Extremely Helpful |  |  | Somewhat helpful |  |  | Not Helpful |

9. Would you be willing to use this pamphlet again?

| ❑_7_ | ❑ | ❑ | ❑ | ❑ | ❑ | ❑_1_ |
| --- | --- | --- | --- | --- | --- | --- |
| Yes, absolutely |  |  | Maybe |  |  | I would rather not |

10. Was your interaction (the shared decision-making conversation) with the patient efficient enough for regular ED care?

| ❑_7_ | ❑ | ❑ | ❑ | ❑ | ❑ | ❑_1_ |
| --- | --- | --- | --- | --- | --- | --- |
| Yes, absolutely |  |  | Maybe |  |  | No, it took too much time |

If there is time, please ask the clinician to answer the following open-ended interview questions, while being recorded. All five would be good, but if not enough time, any combination of them would be useful.

1. What did you think of that interaction?

2. What went well or did not go well?

3. What do you think of the idea of using a nudge?

4. What else could we do to facilitate the use of SDM?

5. What else should the research team know about doing this in real life?
