# Supplementary material for: Feasibility and efficacy of a decision aid for emergency department patients with suspected ureterolithiasis: protocol for an adaptive randomized controlled trial
Source: Trials. 2021 Mar 10;22:201. doi: 10.1186/s13063-021-05140-9 (PMC7944622; doi:10.1186/s13063-021-05140-9)
Supplement: Supplementary file 4 — Additional file 4. Post-encounter patient feedback about clinician data collection forms, 6 pages. [file 13063_2021_5140_MOESM4_ESM.pdf]

Participant Feedback Form

Now we are going to ask you a few questions about today's visit.

These questions will help us know if we are communicating well. Please answer True, False, or Not Sure. If you do not know, please do not guess – please check "Not Sure."

	True	False	Not Sure
1. Kidney stones are caused by crystals that form in the urine.	❑	❑	❑
2. A urine test showing red blood cells in the urine is a sign that you might have a kidney stone.	❑	❑	❑
3. A patient should have a CT every time they have symptoms of a kidney stone.	❑	❑	❑
4. An ultrasound can diagnose gallstones, cysts, and sometimes kidney stones.	❑	❑	❑
5. An ultrasound exposes your body to some radiation.	❑	❑	❑
6. The radiation from a CT scan is equal to the radiation from about 200 X-rays.	❑	❑	❑
7. Radiation increases your future risk of cancer.	❑	❑	❑
8. Having a CT scan will help your kidney stone pass faster.	❑	❑	❑
9. Having a CT scan will usually make your stay in the ED longer.	❑	❑	❑
10. People with kidney stones usually have to be admitted to the hospital (stay overnight).	❑	❑	❑
11. An ultrasound is part of the guidelines for diagnosing kidney stones.	❑	❑	❑

We would like to know about YOUR priorities. If we had the power to make some of the changes listed below, which would be most important to you?

	High Priority	Middle Priority	Low Priority
Decrease the amount of radiation your body is exposed to	❑	❑	❑
Decrease the amount of time you spend in the Emergency Department	❑	❑	❑
Increase you knowledge and understanding of your medical problems	❑	❑	❑
Increase how satisfied you are with your Emergency Department visit	❑	❑	❑
Increase the amount that you are part of decisions about your health care	❑	❑	❑
Decrease the cost of your visit (for you or your insurance)	❑	❑	❑

The following questions are about today's visit

During today's visit…

1. …How much *effort was made* to help you understand *your health issues*? (Circle one number)

No effort at all was made — Every effort was made

1 2 3 4 5 6 7 8 9 10

2. …How much effort was made to *listen to the things that matter most to you* about your health issues? (Circle one number)

No effort at all was made — Every effort was made

1 2 3 4 5 6 7 8 9 10

3. …How much effort was made to *include what matters most to you in choosing* what to do next? (Circle one number)

No effort at all was made — Every effort was made

1 2 3 4 5 6 7 8 9 10

The following questions are about the provider who took care of you (e.g. doctor, nurse practitioner, physician's assistant)

	Strongly Disagree	Disagree	Neutral	Agree	Strongly Agree
The provider cared more about what was convenient for him/her than about your medical needs.	❑	❑	❑	❑	❑
The provider was extremely thorough and careful.	❑	❑	❑	❑	❑
You completely trust the provider's decisions about which medical treatments are best for you.	❑	❑	❑	❑	❑
The provider was totally honest in telling you about all of the different treatment options available for your condition.	❑	❑	❑	❑	❑
All in all, you have complete trust in the provider.	❑	❑	❑	❑	❑

Using any number from 0 to 10, where 0 is the worst provider possible and 10 is the best provider possible, what number would you use to rate this provider? (Provider means doctor, Physician Assistant, or Nurse Practitioner)

<u>Worst Provider Possible</u> <u>Best Provider Possible</u>

0 1 2 3 4 5 6 7 8 9 10

Would you recommend this emergency department to your friends and family?

- ❑ Definitely No
- ❑ Probably No
- ❑ Probably Yes
- ❑ Definitely Yes

Using any number from 0 to 10, where 0 is the worst care possible and 10 is the best care possible, what number would you use to rate *your care during this emergency room visit*? (Circle one number)

Worst Care Possible										Best Care Possible
0	1	2	3	4	5	6	7	8	9	10

For the next question there are THREE response options, so please read all the options before you respond. The question is: Were you as involved in today's decisions as you would have liked to be?

- ❑ Yes, I was as involved as I wanted to be
- ❑ No, I would have liked to be more involved
- ❑ Not applicable, there were no decisions for me to be involved in today

In your opinion, who made the decision about whether or not to get a CT scan today?

- ❑ I made the decision on my own.
- ❑ I made the decision after seriously considering my doctor's opinion.
- ❑ The doctor and I shared the responsibility for making the decision after considering both of our opinions.
- ❑ The doctor made the decision after seriously considering my opinion.
- ❑ The doctor made the decision on his/her own.
- ❑ I don't know.

"Shared Decision-Making" is when your provider explains that there are two medically reasonable options for your care – like two different tests or two different treatments – and then helps you figure out what might make the most sense for YOU.

In your opinion, did "Shared Decision-Making" happen today regarding the TESTS that were done to diagnose your symptoms?

Not at all			Maybe a little bit			Yes, definitely
☐	☐	☐	☐	☐	☐	☐

The following questions are about the discussion you had about your symptoms and your options for care. Please mark the best answer to each of these questions by marking an X in the box you select.

A. How would you describe the **amount of information** about your symptoms and options for care during this visit?

Too little information			Just the right amount of information			Too much information
☐	☐	☐	☐	☐	☐	☐

B. How would you describe the **clarity of information** about your symptoms and options for care during the visit?

Not clear at all			Somewhat clear			Extremely clear
☐	☐	☐	☐	☐	☐	☐

C. How **helpful** was the information about your symptoms and options for care during the visit?

Not helpful at all			Somewhat helpful			Extremely helpful
☐	☐	☐	☐	☐	☐	☐

D. In the future, would you want to get information about other options for care **in the same way** that you got information about your symptoms and options for care during this visit?

No, not at all			Not sure			Yes, for sure
☐	☐	☐	☐	☐	☐	☐

Almost done! The researcher is going to ask you 6 questions about a label. This label is the information on the back of a container of a pint of ice cream.

You have as much time as you need to look for the answer in the label, please take your time.

Research Staff Section
Participant ID: __________
Date: _________________
Initials of RA: ___________
Entered in RedCap: [ ]
Date Entered:_____
Initials of RA doing data entry: ____
