# Supplementary material for: Feasibility and efficacy of a decision aid for emergency department patients with suspected ureterolithiasis: protocol for an adaptive randomized controlled trial
Source: Trials. 2021 Mar 10;22:201. doi: 10.1186/s13063-021-05140-9 (PMC7944622; doi:10.1186/s13063-021-05140-9)
Supplement: Supplementary file 5 — Additional file 5. Use of Healthcare Services. A 4-page healthcare procedures diary. [file 13063_2021_5140_MOESM5_ESM.docx]

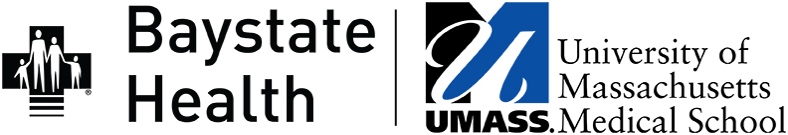


Emergency Department Kidney Stone Study:
Use of Healthcare Services

You were recently enrolled in the ED – Kidney Stone Study. As part of this trial, we would like to understand your use of healthcare services in the 2 months AFTER discharge from the emergency department.

This Diary is for you to keep, in order that you may have a record of events related to your health from the time of your discharge from the emergency department.

In addition, as you may be aware, a study researcher will call you at home in about 14 days and 60 days after you joined the study to ask you about events related to your health.

This Diary will help you keep track of events and answer the questions. If you require additional space to record details of health service use, please use the last sheet.

Please use this diary to record

your trips to any doctors or hospitals

for the next 60 days.

**
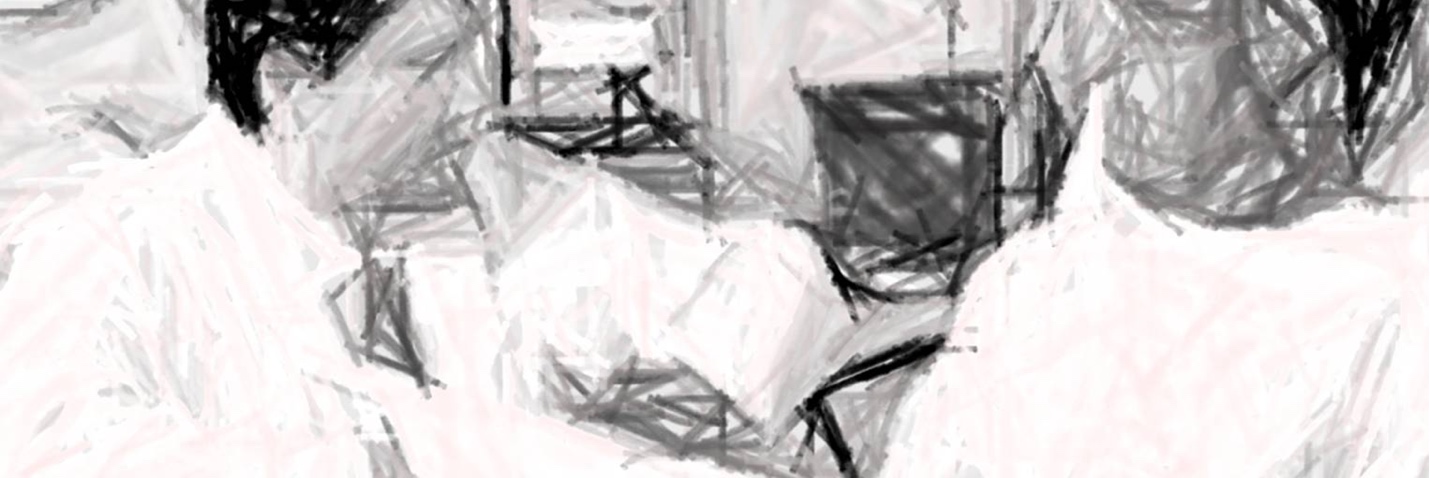
**

Emergency Department Visits

If you visit an emergency department, please write the name of the hospital, reason for the visit and date of each visit. Use one line for each Emergency Department visit.

| Name of Hospital | Reason for Visit | Date of Visit |
| --- | --- | --- |
|  |  |  |
|  |  |  |
|  |  |  |
|  |  |  |

Hospital Admissions

If you are admitted to a hospital, please write the name of the hospital, reason for being admitted and dates of each admission and discharge. Use one line for each hospital admission.

| Name of Hospital | Reason for Admission | Date of Admission | Date of Discharge |
| --- | --- | --- | --- |
|  |  |  |  |
|  |  |  |  |
|  |  |  |  |

Physician Office Visit

If you visit a physician’s office, please write the name of the health care facility, reason for the visit, type of physician and date of each visit. Use one line for each office visit.

| Name of clinic or physician | Reason for Visit | Type of Physician | Date of Visit |
| --- | --- | --- | --- |
|  |  | ☐ Primary Care  ☐ Urologist  ☐ Other: _____________ |  |
|  |  | ☐ Primary Care  ☐ Urologist  ☐ Other: _____________ |  |
|  |  | ☐ Primary Care  ☐ Urologist  ☐ Other: _____________ |  |

Testing

In the next section, we would like you to document any testing that you receive in the 60-days after leaving the emergency department. This testing includes blood tests, x-rays, CTs, or other test you receive. Please indicate the type of test, hospital or clinic where the test was conducted, and date of test.

| Type of Test | Name of Hospital | Date of Test |
| --- | --- | --- |
| ☐ Blood Test  ☐ CT (Computed Tomography)  ☐ Ultrasound  ☐ Other (please describe):__________________ |  |  |
| ☐ Blood Test  ☐ CT (Computed Tomography)  ☐ Ultrasound  ☐ Other (please describe):__________________ |  |  |
| ☐ Blood Test  ☐ CT (Computed Tomography)  ☐ Ultrasound  ☐ Other (please describe):__________________ |  |  |
| ☐ Blood Test  ☐ CT (Computed Tomography)  ☐ Ultrasound  ☐ Other (please describe):__________________ |  |  |

Procedures/Surgery

In the next section, we would like you to document any procedures or surgeries you receive in the 60-days after leaving the emergency department. This includes procedures in the hospital or at an outpatient surgical center, and could include procedures for kidney stones, gall stones, appendicitis, etc. Please indicate the type of surgery, hospital or clinic where you had it done, and the date of the procedure.

| Type of Surgery/Procedure | Name of Hospital or Doctor | Date of Procedure |
| --- | --- | --- |
| ☐ Procedure/surgery for kidney stone:  ☐ Procedure/surgery for: _______________________ |  |  |
| ☐ Procedure/surgery for kidney stone:  ☐ Procedure/surgery for: _______________________ |  |  |
| ☐ Procedure/surgery for kidney stone:  ☐ Procedure/surgery for: _______________________ |  |  |
| ☐ Procedure/surgery for kidney stone:  ☐ Procedure/surgery for: _______________________ |  |  |

Additional Space: Please record any information that did not fit on the previous pagers, or that you think would be useful to researchers.

___________________________________________________________________________

___________________________________________________________________________

___________________________________________________________________________

___________________________________________________________________________

___________________________________________________________________________

___________________________________________________________________________

If you have not heard from the research team one month after your ED visit, it is possible that we are having a hard time getting through to you. Please call us at XXX, text us at XXXX, or email us at XXXX.
