# Supplementary material for: Feasibility and efficacy of a decision aid for emergency department patients with suspected ureterolithiasis: protocol for an adaptive randomized controlled trial
Source: Trials. 2021 Mar 10;22:201. doi: 10.1186/s13063-021-05140-9 (PMC7944622; doi:10.1186/s13063-021-05140-9)
Supplement: Supplementary file 6 — Additional file 6. Data Safety Monitoring Plan and Instructions, 4 pages. [file 13063_2021_5140_MOESM6_ESM.docx]

Supplementary Material 6: Data Safety Monitoring Plan (DSMP)

**Data Safety Monitoring Plan (A) & Instructions (B)**

**A. Data Safety Monitoring Plan**

The following is the DSMP for “Shared Decision-Making for the Promotion of Patient-Centered Imaging in the Emergency Department: Suspected Kidney Stones (ED-Kidney Stone Study)”

As this study is not greater than minimal risk, no Data Safety Monitoring Board is required. This document will instruct the research team and external reviewers regarding how to classify adverse events and interpret stopping criteria.

**Definition of Adverse Events (AE)**

An *Adverse Event* is any untoward medical occurrence, whether or not it is intervention-related.

For this study, AEs are:

1. Admission at time of enrollment or within 30 days,

2. Inpatient surgical procedure (urological and otherwise),

3. Death

As urologic procedures and repeat ED visits are both expected, and collected as part of outcome data, they will not count as AEs, unless patients are admitted to the hospital.

Definition of Serious Adverse Events

For the purposes of this study, hospitalization for pain control or a urologic procedure will not count as a SAE, as hospitalization is an expected outcome in 10% of patients with renal colic. Hospitalization for a non-urologic procedure or antibiotics, anticoagulation, or other medications, admission to the ICU, and death will count as SAEs.

Severity & Relatedness Classification of an Adverse Event

SAEs will be defined as mild, moderate, and severe and will be assessed by two external physician-reviewers (not study staff) for relationship the study intervention (Definitely Related, Probably Related, Possibly Related, and Unlikely to be Related). Degree of relatedness will be determined by assessing the likelihood that the SAE would have occurred if the subject had been randomized to the other treatment arm.

Expectedness

The reviewers will be help determine whether an AE is expected or unexpected. AEs are expected if they are known complications of renal colic (return to the ED, need for procedures, etc) or known complications/ risks that have been explained to the patient (risk of appendicitis if CT not obtained). Examples below.

Examples:

| Event | Classification |
| --- | --- |
| Patient in control group returns with urosepsis and is admitted to the ICU | Moderate SAE, unlikely to be related, unexpected |
| Patient in intervention group returns with *unruptured* appendicitis, is admitted for surgery. No ICU stay. | SAE, mild (unruptured), possibly related, expected. |
| Patient in intervention group returns with ovarian torsion, requires surgery, ovary is necrotic. No ICU stay. | SAE, possibly related, unexpected (if torsion but no necrosis, expected) |
| Patient in intervention group returns to ED with increased pain and fever. Requires admission and urologic procedure. | AE, moderate, expected, possibly related |
| Patient in intervention group returns with retroperitoneal hemorrhage requires IR and lengthy ICU stay | SAE, severe, unexpected, possibly related |

Time period and frequency for event assessment and follow-up

AEs will be solicited at the first and second follow-up as well as by record review. All AEs will be recorded and Serious, Moderate/Severe, and Related AEs will be reviewed by the PI weekly.

As admissions and repeat ED visits with admissions are expected for ~10% of patients, admission alone will not require external review. All other AEs will be reviewed by external reviewers for severity and relatedness.

Reporting procedures

The PI is responsible for AE reporting.

- All deaths and immediately life-threatening events, whether related or unrelated, will be recorded on the SAE Form and submitted to the IRB within 24 hours of site awareness.
- Other SAEs regardless of relationship, will be submitted to the IRB within 72 hours of site awareness.
- As admissions and repeat ED visits with admissions are expected for ~10% of patients, admission alone will not require external review. All other AEs will be reviewed by external reviewers for severity and relatedness.
  - Therefore, the PI will enter all AEs in this form. If SAE+, the clinical course will be reviewed by another physician.

Study halting rules

The study will be reviewed if three “Serious, Moderate/Severe” SAEs determined to be “probably related” are reported. External review will then determine whether the study should continue or be modified.

**B. ADVERSE EVENTS (AE) Instructions**

This page is the instructions for recording of AEs.

**Instructions:**

A. Subject number

B. Description of clinical event including all relevant data and final diagnosis

C. HRDwC (Y/N/unsure): (see definition below)

D. SAE (Y/N): Serious Adverse Events – definition above

E. Severity:

Mild: full recovery anticipated (hospitalization, urology procedure, appendectomy),

Moderate: prolonged recovery (ruptured appy, brief ICU stay),

Severe (ICU stay >72 hours, death)

F. Relatedness:

Definitely Related (if patient was in other group, would definitely NOT have had this outcome)

Probably Related (if patient was in other group, would probably NOT have had this outcome),

Possibly Related (if patient was in the other group, there is a chance they would have had this outcome)

Unlikely to be Related (either group could have this outcome, it has equal probability in both groups)

H. Expectedness:

Expected: known complications of renal colic (return to the ED, need for procedures, etc) or known complications/ risks that have been explained to the patient (risk of appendicitis if CT not obtained, risk of cancer being discovered on subsequent CT).

Unsure:

Unexpected: not a complication of renal colic, and not a generally described risk of delaying CT

**Safety Outcomes:**

HRDwC:

High risk diagnosis with complications (HRDwC) are being recorded as part of safety outcomes for the study. (abdominal aortic aneurysm with rupture, pneumonia with sepsis, appendicitis with rupture, diverticulitis with abscess or sepsis, bowel ischemia or perforation, renal infarction, renal stone with abscess, pyelonephritis with urosepsis or bacteremia, ovarian torsion with necrosis, or aortic dissection with ischemia.^10^)

Alternative Diagnoses

Important and Emergent Alternative Diagnoses will also be recorded, and time of diagnosis (days after initial visit) will be recorded. However, these will not be classified as AEs unless they meet the above criteria.

Alternative diagnoses: *Something that may require follow-up but does not receive immediate treatment at ED visit*

| Malignancy or new mass | Epiploic appendigitis |
| --- | --- |
| Ovarian mass needing follow-up | Ovarian cyst, hemorrhagic or otherwise |
| AAA less than 5cm | Biliary colic |
| Splenomegaly | Iliac aneurysm, unruptured |
| Mesenteric Adenitis |  |

Emergent Alternative Diagnoses: *Receiving intervention or action at the time of diagnosis (procedure, antibiotics, anticoagulation, etc)*

| Appendicitis | Colitis | Intra-abdominal abscess |
| --- | --- | --- |
| Diverticulitis | Enteritis | Intra-abdominal thrombus |
| Cholecystitis | Ovarian Torsion (not simple or hemorrhagic cyst unless OR needed) | Bowel perforation |
| AAA >5cm | UTI/cystitis/Pyelonephritis | Renal infarct or hemorrhage |
| SBO | Urosepsis | Retroperitoneal hemorrhage |
| Pneumonia | Aortic dissection |  |
| Pancreatitis |  |  |

See Instructions for filling out this chart. Participants should be entered in this chart if:

1. Admission at time of enrollment or within 60 days,

2. Inpatient surgical procedure (urological and otherwise)

3. Death

| A. Subject number | B. Description of event including final diagnosis | C. HRDwC (Y/N/unsure) | D. SAE (Y/N) | F. Severity (mild, moderate, severe) | G. Relatedness: (Definitely Probably Possibly Unlikely to be Related) | H. Expectedness: (+exp/unc/unexp) |
| --- | --- | --- | --- | --- | --- | --- |
|  |  |  |  |  |  |  |
|  |  |  |  |  |  |  |
|  |  |  |  |  |  |  |
|  |  |  |  |  |  |  |
|  |  |  |  |  |  |  |
|  |  |  |  |  |  |  |
|  |  |  |  |  |  |  |
